# Smart Specialisation Strategies and regional knowledge spaces: how to bridge vision and reality

**DOI:** 10.1080/00343404.2024.2355985

**Published:** 2024-06-06

**Authors:** Keungoui Kim, Chiara Ferrante, Dieter F. Kogler

**Affiliations:** a School of Applied Artificial Intelligence, Handong Global University, Pohang, South Korea; b Fraunhofer Institute for Systems and Innovation Research (ISI), Karlsruhe, Germany; c Spatial Dynamics Lab, University College Dublin, Dublin, Ireland; d Spatial Dynamics Lab, School of Architecture, Planning & Environmental Policy & Insight Centre for Data Analytics, University College Dublin, Dublin, Ireland

**Keywords:** Smart Specialisation Strategies (S3), policy design and evaluation, regional knowledge spaces, European Union, regional research and innovation policy, O31, O38, R11

## Abstract

Smart Specialisation Strategies (S3) are implemented across European regions. However, investigations into whether S3 initiatives adequately match local knowledge capabilities are very scarce. This work analyses to what extent S3 policies are coherent with the local knowledge space of 164 European regions, respectively. We show to what extent regional S3 policies target ‘central’ technologies, and to what degree S3 policies also target ‘potential’ sectors of knowledge production in specific regional settings. Our findings provide a solution for how S3 policies could be designed in the future to overcome the gap between S3 vision and the reality of constraints in regions.

## 1. INTRODUCTION

Since the introduction of Smart Specialisation Strategies (S3) within the European Union Cohesion Policy 2014–20 framework, and its ‘ex-ante conditionality’ set out in the European Structural and Investments Funds 2014–20 directive that essentially makes it a prerequisite to receive funding from the European Regional Development Fund (European Commission, 2017), S3 policies and actions have been implemented across European regions with the aim to diversify investments in new economic domains and trigger regional economic development. The S3 initiative was designed as a new place-based approach for regional innovation policies, overcoming the ‘one-size-fits-all’ approach to industrial policies (Barca, 2009). Regions, as government and institutional bodies, oversee the identification of new economic and technology domains which are likely to develop in the future, following an ‘Entrepreneurial Discovery Process’ (EDP), which relies on the combination of horizontal/institutional interventions and the application of the vertical/entrepreneurial identification of the sectors to be prioritised (Foray, 2015, 2019; Foray et al., 2009). The idea behind the S3 concept is to implement targeted policies towards innovative sectors, through the strengthening of existing regional economic structures as well as to diversify regions’ economic activities into promising areas, which in turn will help them become more competitive in the future (Morisson & Pattinson, 2020). Although the literature on S3 and its theoretical foundations have widely flourished in the past years, only recently a limited number of studies have started to assess the effectiveness of its implementation. More critically, a more nuanced discussion on whether Smart Specialisation policies that are already implemented in regional economies actually match their local knowledge base as indicated by advanced measures is still missing (Marrocu et al., 2023; Rigby et al., 2022). The lack of enquiries into how current regional S3 actions align with local knowledge production capabilities poses significant challenge for policymakers, practitioners as well as entrepreneurs. Above all, it creates a situation where it is not possible to determine whether a relevant policy is designed properly to address its original purpose, and more specifically it prohibits relevant stakeholders from accessing practical and easy-to-use tools that would enable them to anticipate and evaluate both existing competitive advantages and future segments of potential growth in their respective regional settings. In this process, a region’s ability to identify its core innovative capabilities, in conjunction with possibly related activities that hold future potential in their knowledge space, are crucial. This in turn will allow them to aid regional specialisation and diversification, resulting in productivity gains and associated regional economic growth (Rocchetta et al., 2022). In addition, it will also support efforts towards building regional synergies, as well as minimise the risks of technological lock-in (Boschma, 2017; Kogler, 2017).

In order to address current shortcomings and to offer an approach that could potentially provide essential insights into the development of S3, the present investigation explores to what extent current S3 policies are coherent with the local technological knowledge space of 164 European regions (Kogler et al., 2013). The identification of the regional knowledge space plays a fundamental role in recognising the knowledge domains in which each region is already specialised, and how all domains across the knowledge production spectrum relate to each other (Kogler et al., 2013). Subsequently, insights into place-based knowledge then enables regions to direct policy efforts towards particular segments of the local knowledge space, which in turn avoids the implementation of a universal policy strategy for all regions that is most likely ineffective and doomed to fail (Tödtling & Trippl, 2005). Essentially, the aim is to illustrate where regional Smart Specialisation policy actions also coincide with local knowledge capabilities, that is, that a policy is actually targeting knowledge domains where a region has high potential and thus can leverage its strengths. We achieve this objective from two different perspectives. We explore how much regional-specific S3 policies target ‘central’ technologies as indicated by their position in the respective regional knowledge space. Further, the investigation highlights to what degree S3 policies also target ‘potential’ sectors of technological knowledge production in a specific regional setting. For clarity, the investigation is centred around to key questions:

- Are smart policy priorities directed at ‘central’ knowledge domains in regional knowledge spaces?
- Are S3 policy priorities targeting knowledge domains that hold the ‘potential’ for further regional diversification opportunities?

By addressing these two questions the study will offer ample insights into whether a regional S3 has been properly designed to address a region’s ambitions regarding the future of its local knowledge structure, and in particular if there is a gap between the regional Smart Specialisation policy agenda in relation to present and potential local knowledge production capabilities. Employing well-established network and relatedness measures, the main objective and contribution of this work is to provide a more accessible tool for policymakers that will allow them to select their regional priority domains, and pursue strategies that either aim to strengthen existing patterns of specialisation and/or high potential domains in their efforts to design and implement effective S3 polies that align with real local knowledge expertise.^1^

The paper is structured as follows. The next section provides an overview of the theoretical arguments and some of the relevant studies associated with this research agenda. Section 3 describes the dataset that will be utilised along with the methodological framework that will be implemented in order to answer the two specific research questions outlined above. Section 4 highlights the results of the two frameworks analyses and offers a detailed interpretation. Section 5 outlines the main policy implications from which one can draw the present investigation. The final section concludes.

## 2. THEORETICAL UNDERPINNINGS AND RELEVANT STUDIES

### 2.1. S3 and criticisms thereof

In their pursuit to implement effective S3 actions, regions should identify and prioritise their strong or promising innovation, entrepreneurial or research activities (Morisson & Pattinson, 2020). The ‘Entrepreneurial Discovery Process’ (EDP) is at the heart of this objective: the discovery of new activities can generate new opportunities and translate into benefits not only for regional technological innovation but also for the whole regional economy (Foray, 2014). This process can be pursued through the transition of an existing activity from a declining market to a newly developed market; through the modernisation of used technologies; through diversification in both emerging and existing activities; or by the radical foundation of a new activity (Foray, 2014). The fulfilment of the EDP implies the existence of proper capabilities and the skills to reach this scope, and an underlying institutional structure able to run this process efficiently. Indeed, when assessing the potential development of a regional knowledge space, it is necessary to account for the universal entrepreneurial opportunities that reside in that region, which can be very different from those in other regions. In this regard, it is not only the purely innovative entrepreneurship potential, albeit of utterly importance, that drives particular development outcomes. In tandem, it is also place-based leadership and institutional entrepreneurship that are intrinsic to a locality and that provide further opportunities and capabilities to enhance regional discovery processes which are equally relevant in order to successfully pursue new avenues of technological change and growth. Thus, it is this ‘trinity of agency’ (Grillitsch & Sotarauta, 2020) that determines what place-based development opportunities might exist at a particular locality in the first place, and then also the opportunities in terms of how this can actually be translated in competitive advantages. Overall, although these particular qualities of a place might be difficult to evaluate and to measure, it is important to account for these and the inherent heterogeneity across regions and countries.

The institutional framework of regions is also essential for the initial implementation of S3 policies. From the onset, scholars have mooted several criticisms concerning the concept and highlighted how S3 implementation might be problematic, especially in terms of regional institutions that are frequently not equipped with the right skills and knowledge (Hassink & Gong, 2019). Effectively, low quality regional governance and weak institutional structures pose a real challenges for an effective implementation of regional S3. Furthermore, referring to the S3 policy design directly, it has been noted that the application of well-established theoretical concepts or empirical methods is frequently missing, with a widespread preference for designing policies through informal methods or by simply following the neighbouring region’s strategy (Balland et al., 2019; Foray et al., 2011; Morgan, 2015; Santoalha, 2019).

Essentially, regions frequently are not able to manage the political interests and the social dynamics at play, and they experience difficulties in providing an institutional framework for the development of new innovative activities and the realisation of the EDP (Sotarauta, 2018). Grillitsch (2016) also points out how place-based policies, if not supported by well-functioning institutions, might bring about problems such as corruption episodes, risk of lock-in and pressure from local groups with a vested interest. Weak regional structures and the lack of skills and capabilities also limit the creation of synergies and networks, hampering the proper identification of emerging and innovative sectors (Benner, 2020; Capello & Kroll, 2016; Hassink & Gong, 2019). Therefore, institutional quality is crucial to the development of place-based innovation policies, especially for peripheral and lagging regions. The S3 concept was designed to address less-developed regions to give them the chance to build or develop competitive advantages in their pool of activities (Foray, 2014). However, it is lagging regions in particular that frequently have to rely on weak governance structures, which combined with development constraints, hampers their effectiveness in terms of policy implementation (Boschma, 2014; McCann & Ortega-Argilés, 2015; Rodríguez-Pose et al., 2014). Thus, S3 also require innovation in the policy environment for its correct and effective implementation, and a lack thereof will result in innovation and policy design processes that are likely to fail from the onset (Borrás, 2011; Moodysson et al., 2015). The effective operationalisation and implementation of S3 requires a ‘place-based policy framework’ that considers and creates a policy design around strong and emerging sectors in each regional setting (Barzotto et al., 2020), and only then will the concept actually also fulfil its anticipated potential.

### 2.2. Smart Specialisation and Evolutionary Economic Geography

In an attempt to fill the gap between theory and implementation, scholars have produced a multitude of studies to link S3 to relatedness and complexity concepts and, only recently, more applied studies that assess the coherence between policies and S3 objectives (Marrocu et al., 2023; Rigby et al., 2022). In this regard, a region’s pre-existing capabilities and its ability to exploit them for enhancing specialisation and diversification into new economic domains, is considered highly important; frequently referred to as the concept of related diversification (Neffke et al., 2011). The notion that knowledge creation processes are path-dependent and evolutionary, and that regional innovation and development trajectories hinge on pre-existing knowledge, is firmly grounded in the Evolutionary Economic Geography (EEG) paradigm (Dosi, 1982; Grabner, 1993; Kogler, 2015; Kogler et al., 2023a). In this context, it is acknowledged that the presence of particular knowledge domains and their respective connectedness, which will vary from place to place, strongly influences both the potential and the limits for structural evolution over time; this is also highlighted and discussed in early S3 policy initiative documents (Foray et al., 2009, 2011). Connectedness in this context refers to the recombinant power of knowledge in a Schumpeterian sense (Schumpeter, 1939), where most novel knowledge is not original per se, but the result of new combinations of knowledge domains that might have already existed in separation previously. Building on these ideas, while translating them into a spatial context, it is the concept of regional knowledge spaces (Kogler et al., 2013) that then prescribes a methodology capable of capturing and quantifying local knowledge domains, their respective relationships across different areas of the overall knowledge pool, and how this might also impact on the capacity to generate and accumulate recombinant knowledge outcomes over time. Following this rationale, a number of studies have tested the approach in order to investigate to what extent the relatedness of knowledge domains contributes to technological change (Boschma et al., 2015; Rigby, 2015) and the evolution of regional specialisation (Kogler et al., 2017), and in addition as a tool to evaluate the impact of regional knowledge spaces on productivity and growth (Rocchetta et al., 2022), and economic resilience (Toth et al., 2022), amongst others. The theories behind recombinant knowledge production and associated related diversification, combined with the applied knowledge space methodology, provide a framework for generating essential intelligence for place-based S3 policy design, and of equal importance, offer an approach that will test if S3 actions that are already implemented are in fact geared towards specific local knowledge capabilities with the potential to spur future regional innovative outcomes.

Furthermore, a more applied strand of the relevant literature has recently begun to assess the coherence between the sectors of a particular regional policy implementation and S3 objectives, as well as between specific S3 policies and the link to regional specialisation patters. A number of studies focus on the assessment of the technological development status of European cities and regions and attempt to develop S3 policy frameworks built on the concepts of relatedness (Whittle & Kogler, 2019) and complexity (Deegan et al., 2021) that could potentially assist in identifying more centralised regional diversification strategies. Among these, Balland et al. (2019) find that relatedness has a positive effect on technological diversification within regions: regions with highly related technologies to the local knowledge space tend to diversify and specialise in more complex technologies, triggering their competitiveness and economic growth. Similarly, Rigby et al. (2022) point out how cities and regions that diversify their knowledge cores into related and more complex technological fields are associated with better economic performance. The shortfall of these studies is that they frequently only focus on lager cities and disregard more peripheral and lagging regions. These regions were of particular interest to the original architects of the S3 policy framework and need to be included if we consider S3 as a general and universal innovation policy approach. Furthermore, these studies frequently only provide aggregate relatedness and complexity measures for entire regional economies, which in turn yields very predictable results, that is, larger cities operate in more knowledge domains, these are by definition then more related, and subsequently this results in higher complexity values being present in those innovative centres. Nevertheless, these studies provide new conceptual frameworks that could improve the identification of new technology trajectories and the diversification process in more general terms (Hidalgo, 2023).

Another set of relevant studies takes a more direct approach by focusing on empirical analyses that directly assess the regional implementation of the S3 in Europe, and explore how regions shape their choices for Smart Specialisation policies and their actual effectiveness for the regional technological development. Key studies include Marrocu et al. (2023), which show that most of the regions do not choose sectors for S3 in which they have a competitive advantage or potential for diversification. In summary, they identify four different trajectories on how regional S3 have been designed and are currently implemented, and thus detect a considerable heterogeneity across and within countries towards how the S3 framework is actually exercised. Furthermore, they find a close association between S3 choices and institutional quality, as only regions with a high quality of local government tend to choose sectors for Smart Specialisation policies in which they actually possess current pattern of specialisations or that are related to their existing capabilities. Similar, Di Cataldo et al. (2021) find that S3 policies are weakly linked to a region’s characteristics and that only territories with a high quality of government have chosen more realistic strategies. The findings of another significant study, which employs a relatedness and complexity framework, by Deegan et al. (2021) shows that regions tend to gravitate towards more complex sectors when selecting policy priorities, although without any consideration of their local embeddedness as indicated by relatedness measures. Thus, priorities are chosen independently of their actual position in the regional knowledge space, which in turn lacks a vision of potential synergies and essential complementarities that might be required for a successful economic diversification process to take place. Similar, Gianelle et al. (2020), who assess the coherence of policy decisions surrounding S3 objectives, find that most of these decisions target a set of priorities without a specific rationale, compromising the effectiveness of the policy initiative itself.

Based on these insights, it is obvious that regional decision- and policymakers are in dire need of further guidance in order to ensure the effective design and operationalisation of S3 actions in their respective jurisdictions. In particular, there is a need for more accessible and user-friendly tools that will enable them to select among their already central and/or potential technologies in the quest to design S3 policies that will result in measurable impact. Building on the strand of existing literature, and focusing directly on an assessing S3 implementation, the goal of our investigation is twofold. The goal is to develop and highlight a framework that allows for the assessment of the current state of S3 strategies across European regional economies. For explanatory and illustrative purposes, three specific, highly representative, regional examples will be highlighted. These examples will provide the insights and guidance necessary to take full advantage of all the indicators that will be accessible through the online tool that we have developed.^2^

The second contribution of this study is to provide an accessible and user-friendly online tool that offers a number of established key indicators on all present technological knowledge domains across most European regions, something which in turn can be easily utilised as input into the development of future generations of European regional S3 policy initiatives. Rather than having to rely on aggregate measures that are commonly presented in the relevant literature, the rKnowledge online dashboard offers four distinct and established knowledge space indicators for each knowledge domain that is present across about 700 European metropolitan and non-metropolitan regional economies. The dashboard enables policymakers and practitioners to directly evaluate the overall opportunities in a given knowledge space, but also to see the specific potential each knowledge domain might hold in their respective region.

In summary, the objective of the subsequent sections is to investigate to what extent currently employed regional S3 policies target ‘central’ regional knowledge capabilities and domains that hold a high ‘potential’ for future diversification of the regional knowledge space.

## 3. DATA AND METHODOLOGY

**Figure 1.**
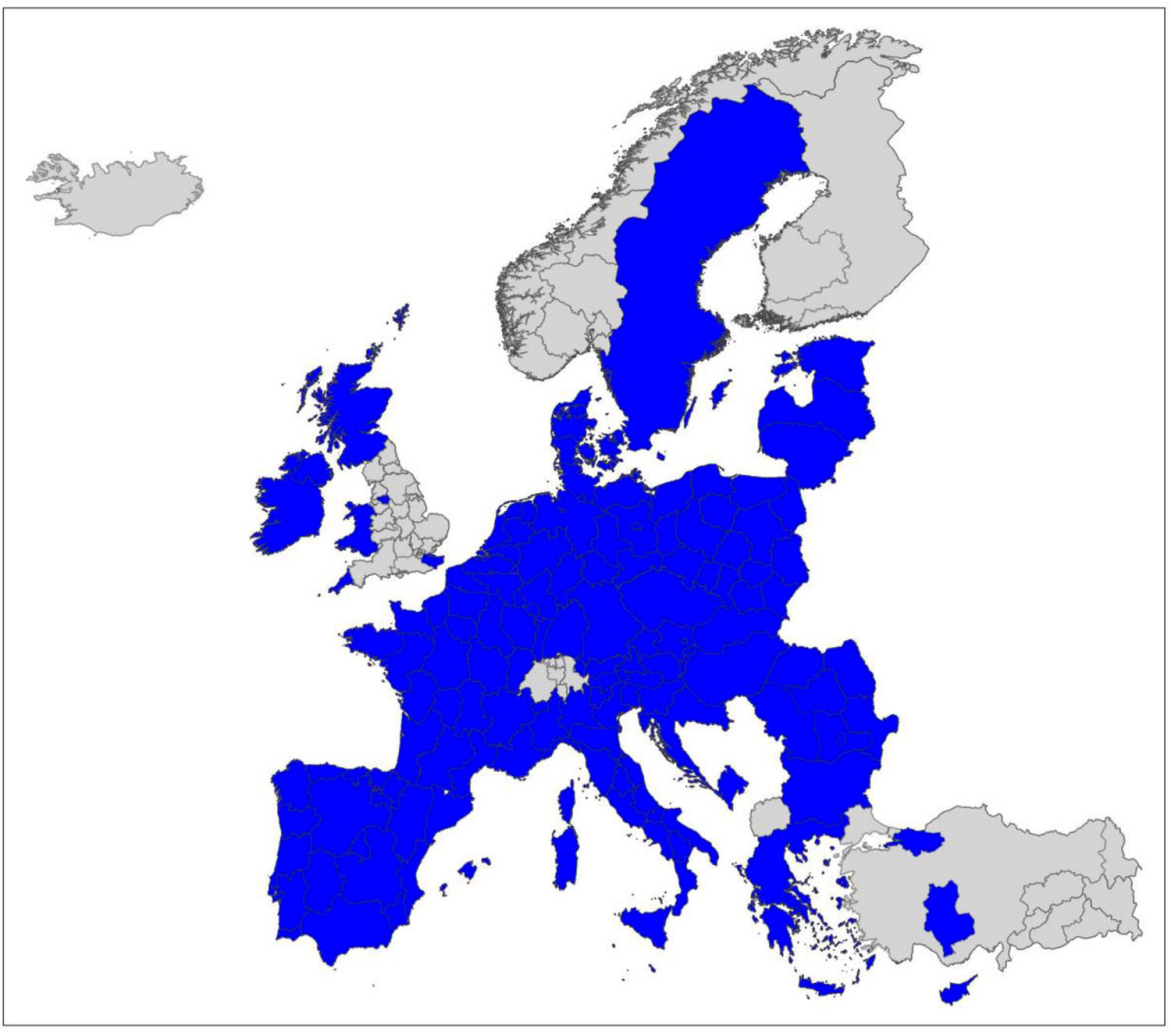
Smart Specialisation policy regions.

The data utilised in this study and the development of our regional S3 online tool have been derived from two sources: the European Patent Office (EPO) PATSTAT database, which has been employed in similar studies (e.g., Kogler et al., 2022; Rigby et al., 2022); and the S3 platform of the European Commission, which provides information on each regional S3 action, including sectoral focus.^3^ The final database covers the 2011–15 period and features at total of 237,192 patents across 164 regions. It should be noted that S3 actions have been implemented at various spatial levels across European countries, including national and NUTS-1−3 territorial levels. For the purpose of the present investigations, S3 actions designed at NUTS-3, for example, in Finland or Sweden, have been aggregated to the NUTS-2 level that also corresponds to the most prominent territorial level across all S3 actions. This then resulted in a total of 133 regions at the NUTS-2 level. S3 defined at the NUTS-1 level were left unchanged but only applied to a total of 31 regions. The few, mostly small, countries where S3 is implemented at the national scale were excluded from the analysis. All regions considered in the analysis that follows are highlighted in Figure 1; while Appendix A in the supplemental data online provides an overview of these regions and their associated NUTS codes in tabular form.

To build the knowledge space for each region under investigation and to subsequently enable the analysis that tests to what degree S3 policy actions are actually directed at ‘central’ and ‘potential’ knowledge domains, it was necessary to link the regionalised PATSTAT database with the data derived from the S3 platform. This merge of the two regional datasets is based on their respective classification codes: the Cooperative Patent Classification (CPC; four-digit level) which lists the relevant technological knowledge domains that contributed to the development of an invention listed on each individual patent document; and the Nomenclature of Economic Activities (NACE; two-digit level) classification of economic activities as defined for each regional S3 policy implemented. Following the Algorithmic links with Probabilities (Lybbert & Zolas, 2014) approach, the probability assigned to each NACE code to fall into particular CPC subclasses is then assigned. The direct link of inventive capacities to economic activities, that is, the CPC to NACE classification concordance, subsequently enables the assessment of how a particular S3 policy implemented in a region matches its local knowledge base, which is captured by the share of the patenting activity by local inventors in a particular class.

**Figure 2.**
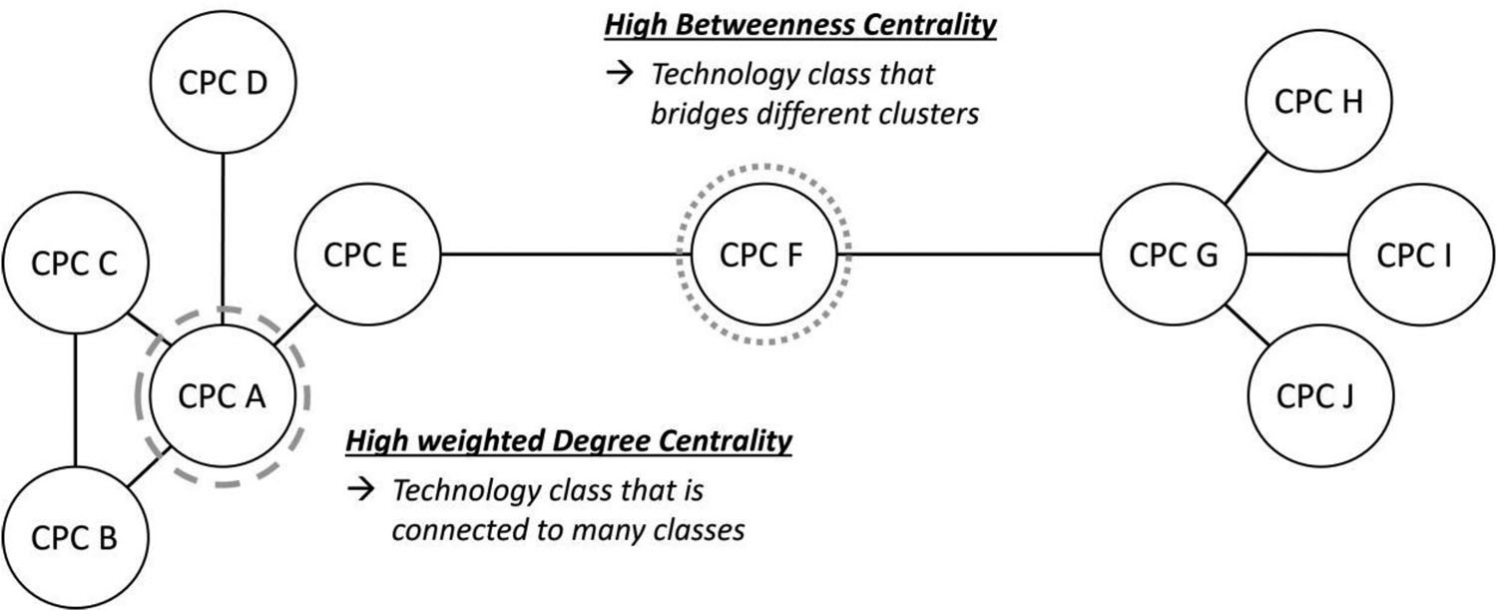
Weighted degree centrality and betweenness centrality. Note: CPC, Cooperative Patent Classification.

In order to answer the two research questions stated previously, two analyses frameworks will be utilised. Both frameworks build on the knowledge space methodology (Kogler et al., 2013, 2017), which is essentially a regionalised CPC co-occurrence network pertaining to the local production of technological knowledge. In that network, CPC classes are the nodes and thus indicate the relative size of a particular knowledge domain, while the edges represent the frequencies of how often two CPC classes are combined on single inventions and thus indicate the recombinant potential of certain classes. In the first analysis framework we take advantage of established network centrality indices to measure how ‘central’ a particular knowledge domain, that is, CPC class, is in a regional knowledge space. Specifically, we measure the weighted degree centrality to capture a domain’s connectivity in the network, and then also the betweenness centrality to gain insights regarding a domain’s brokering and linking powers in the network. In other words, the weighted degree centrality measures how frequently a particular CPC class has been used across different patents, which in turn implies that if a technology class is highly utilised in conjunction with other classes in single inventions, it is also more likely to generate more, and also more diversified, inventive outputs due to its high connectivity. The weighted degree centrality is calculated by counting the weight of edges (Kim et al., 2018, 2019; Kogler et al., 2022; Lee & Kim, 2018). The betweenness centrality then indicates to what degree a particular knowledge domain acts as a broker in the network, that is, how well it connects different clusters of technologies. A highly brokering technology has the potential to create bridges between heterogeneous clusters of technologies within the network (Kim et al., 2018, 2019; Lee & Kim, 2018). Figure 2 illustrates an example of a highly connected (CPC A) and a highly brokering (CPC F) technology class.

The second analysis framework also employs two established measures derived from the analysis of regional knowledge spaces. One pertains to the degree of specialisation of a particular knowledge domain as indicated by the domain’s revealed comparative advantage (RCA). The RCA, also frequently referred to as the location quotient, is computed by dividing the share of a given CPC class in a region by the share of that CPC class across all regions (Balassa, 1965). The sectors that take an RCA > 1 are considered to have a comparative advantage in that region, that is, they are frequently labelled to be ‘specialised’ in the relevant literature (Kogler et al., 2017). The second measure refers to the relatedness density of a particular knowledge domain present in a region. The measure essentially indicates the proximity of a given technology to the existing set of all present CPC classes that are specialised, that is, having an RCA > 1, in a focal region (Boschma et al., 2015; Hidalgo et al., 2007). In other words, the relatedness density then indicates the degree to which a technology class is embedded in the local knowledge space, and by extension how well it utilises the capabilities available in the region for recombinant knowledge production. Higher values of relatedness density imply that a technology class has a high ‘potential’ to be successful and generate growth through diversification (Boschma et al., 2015).

## 4. SMART SPECIALISATION AND LOCAL KNOWLEDGE SPACES

In this section, the findings of the two frameworks that have been introduced above are illustrated and discussed. These two approaches provide two different tools that might offer ample assistance in the identification of relevant sectors and areas that should be prioritised to trigger regions’ innovative potential and associated growth, as envisioned in the S3 policy context.

### 4.1. Are smart policy priorities directed at ‘central’ knowledge domains in regional knowledge spaces?

The first framework provides the setting to discuss whether S3 policies that are presently implemented are actually designed to target local technological knowledge capabilities that are central to regions’ knowledge spaces. Following a knowledge recombination perspective, one can adopt the view that local technologies that are more amendable to recombinant knowledge production, that is, they are frequently used in conjunction with other technologies in the development of novel products and processes, as well as those that hold the potential to link heterogenous clusters of technologies within a particular knowledge space, are those that should be attributed special attention.

**Figure 3.**
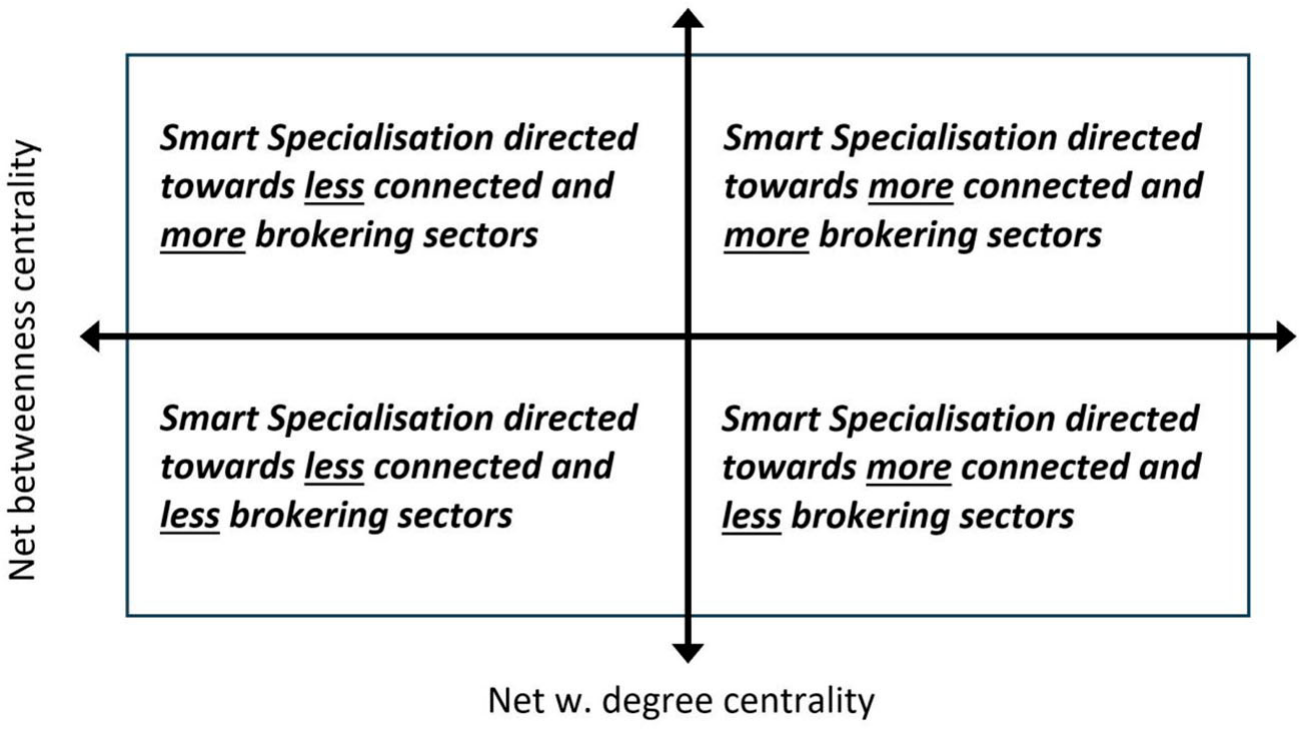
Framework 1: Concept of net weighted degree centrality and net weighted betweenness centrality in the context of S3 priorities.

Accepting these notions, it is then possible to develop a two-dimensional framework that features the net weighted degree centrality and net betweenness centrality measures for each technology, and subsequently to develop a summary indicator encompassing all technologies present in each locality. In a further step, it is then also possible to separate those technologies that are directly addressed by an implemented S3 policy versus those that are not. Figure 3 provides a schematic of this two-dimensional framework along with the quadrants that highlight four specific S3 focus areas as they pertain to the general approach that might be pursued in a particular regional context. In this regard, and for each region in the present investigation, the net values of both network centrality measures are calculated by taking the average of the subtraction of the S3 directed towards sectors versus those where S3 is not directed towards sectors. Essentially, the two network measures of technologies that overlap, or coincide, with the priority areas outlined in a regional S3 policy are set against the measures of those technologies in the region that are not covered, that is, they do not overlap, in the same policy document. In cases where both summarised network measures display a positive sign, this then indicates that the S3 policy in that region is geared and implemented mainly towards knowledge domains that are more connected and more brokering when compared to the technologies that are not covered in the policy action.

**Figure 4.**
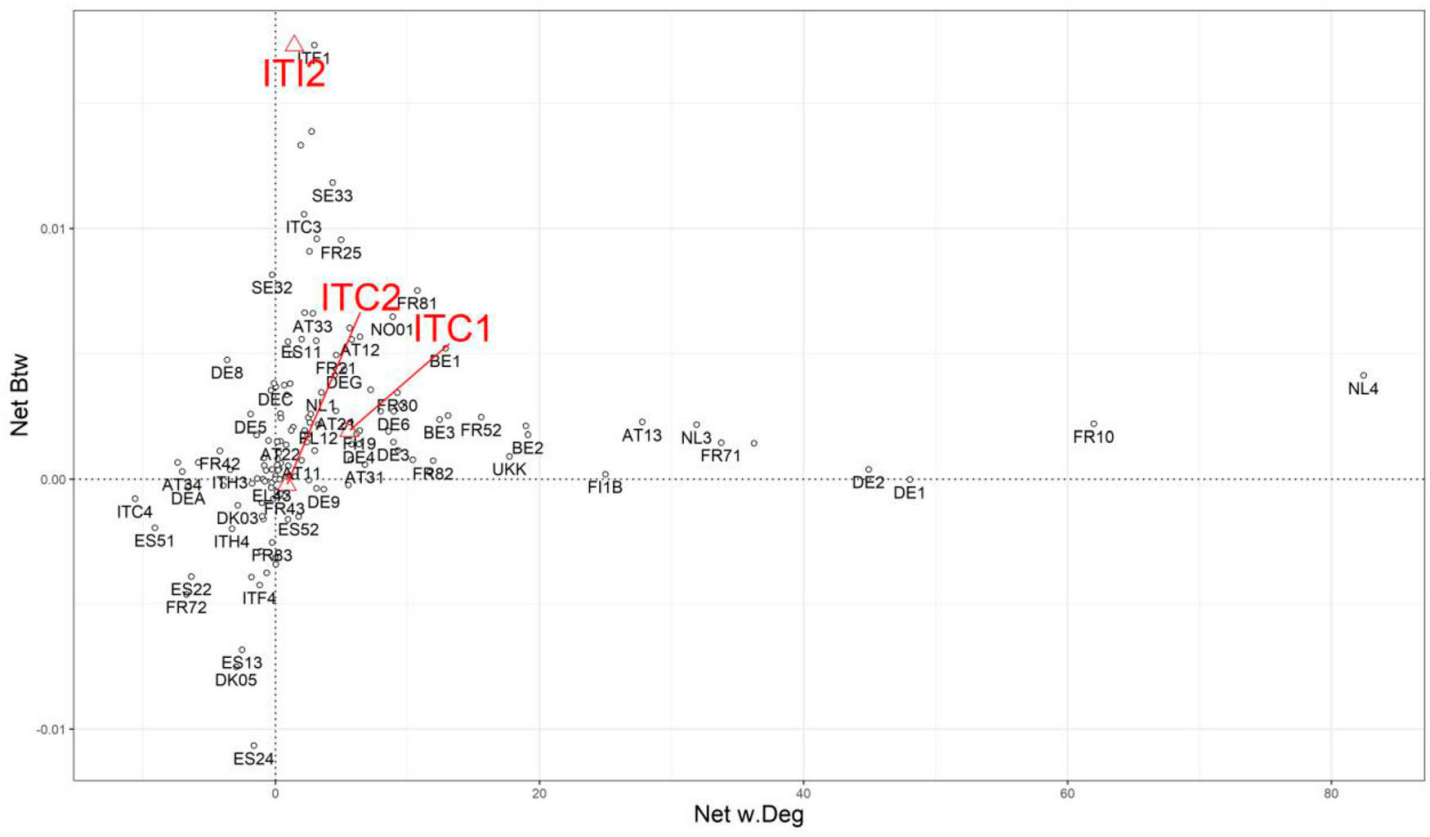
Framework 1: Net weighted degree centrality and net betweenness centrality of Smart Specialisation and knowledge space. Note: The three particular regional cases that will be discussed in detail in the subsequent section are highlighted in this first framework.

The results for all regions in our sample according to this framework are plotted in Figure 4. Regions in the first quadrant are in line with the ideal assumption that S3 actions in those places are indeed focused on ‘central’ sectors in the regional knowledge space, with both net measures being positive. Among them, we see the Brussels, Flanders and Wallonia regions (BE1–3), the Ile-de-France (FR10), amongst others. However, those regions with one of the values very close to zero might still have ample opportunities to improve their position, for example, by becoming more brokering as it is the case for the Bayern region (DE2), or more connected in the case of the Abruzzo region (ITF1). In the second quadrant, regions are partially doing well in having implemented S3 polies that target their more brokering sectors in their regional knowledge space, but certainly could improve their position by also addressing technologies that are well connected, for example, the DE8, DE5, FR42 regional economies. In the third quadrant, regions have implemented S3 policies that target less important sectors in their respective knowledge space, with both values of net betweenness and degree centrality being in the negative. Thus, regions such as Lombardy (ITC4), Cataluna (ES51) or Aragona (ES24) might reconsider the priority areas in their S3 policies towards more connected and more brokering technologies in order to trigger diversification opportunities that are less likely to materialise in the areas of their knowledge space that is currently targeted. Regions that are situated in the fourth quadrant, similar to those already mentioned in the second quadrant, only perform well on one indicator, that is, their S3 policies are aligned with ‘central’ sectors in the knowledge space that constitute well connected knowledge, while technologies with brokerage abilities are neglected. In general, one can observe that most regions fall within either the first or third quadrant, which indicates that currently implemented S3 policy actions across European regional economies are either designed to take advantage of ‘central’ knowledge domains that hold the potential for recombinant knowledge production and regional diversification opportunities, or they are quite misaligned with local strengths and competencies that reside in the relevant local knowledge space.

In general, the framework followed in Figure 4 provides regions with concrete underlying measures to assess their current knowledge space, including information on each knowledge domain if looked at a disaggregated level, and indicates via established network measures which sectors should be prioritised in order to leverage S3 policy-related investments. Pending on a region’s stance towards S3 policy design, several potential avenues can then be pursued, including the option that would address both connecting and brokering knowledge domains, in order to create an innovative environment characterised by more connected and related clusters of sectors, and in turn building a structured approach to enable the much sought after entrepreneurial discovery process. As also demonstrated by Kogler et al. (2022), awareness of the network properties of the local knowledge space can be crucial in determining the regional development towards high-potential technological sectors. Addressing Smart Specialisation to those sectors that match the regional knowledge capabilities and its network structure can trigger further innovation and development in related industries (Neffke et al., 2011). To address those concerns further, and grasp and understand the different regional innovation strategies that are currently pursued across regional economies, we now move to answer the second research question that guides the present investigation.

### 4.2. Are S3 policy priorities targeting knowledge domains that hold the ‘potential’ for further regional diversification opportunities?

**Figure 5.**
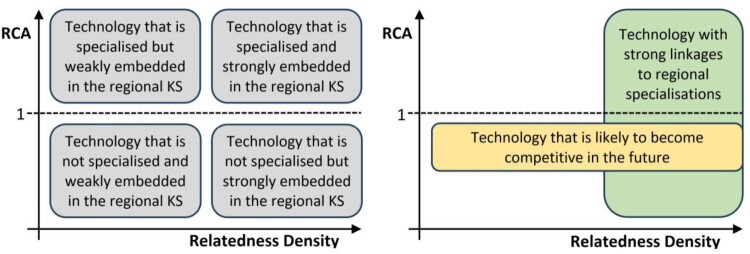
Framework 2: Revealed comparative advantage (RCA) and relatedness density. Note: KS, knowledge space.

Regions might adopt different innovative strategies conditional on their previous and existing economic structure and their innovative capabilities and their organisational strengths (Boschma, 2017; Trippl et al., 2020). However, it is not clear if current S3 policies that are implemented across European regional economics are directed towards either ‘potential’ or already ‘specialised’ local technologies. In order to investigate this further, we apply the RCA and the relatedness density measures outlined above and that are frequently employed in similar studies (Boschma et al., 2015). Figure 5 develops a conceptual framework that sets to measures in the context of the relative potential they offer along the two dimensions. Initially, we distinguish technologies by their RCA, depending on whether they are above or below 1. Subsequently, we distinguish between those technologies that have a higher level of relatedness density, which are the nodes in the local knowledge space that are more embedded opposed to those characterised by lower relatedness density levels.

Pending on the relative position in this two-dimensional framework one can prescribe a typology of four distinct node characteristics unique to each knowledge domain in a local knowledge space (see the left-hand side of Figure 5). However, in the present investigation we are interested in the approach taken by the regions in their implementation of S3 policies within this proposed framework. For example, regions might choose to implement policies in already specialised and embedded technologies to strengthen their existing patterns of specialisation. Nevertheless, such an approach might impede potential diversification opportunities in terms of nodes in the knowledge space that are only weakly specialised but at the same time demonstrate a high ‘potential’ due to their relative strong embeddedness as indicated by high relatedness density values.^4^ On the right-hand side of Figure 5 we further refine the original framework into two important considerations regarding knowledge domains that hold the potential to initiate structural change and diversification in the local knowledge space, that is, nodes that are already strongly embedded, but not yet specialised in the network.

While the framework points towards the traits of knowledge domains regional S3 actions should target to maximise the potential that resides in current knowledge space, the actual decision of what strategy to pursue of course strongly depends also on a region’s institutional capabilities, its level of regional development, and its pre-existing technologies and patterns of specialisation in general.

To highlight the variations in the approaches taken in regional S3 implementation strategies, we now turn to three specific regional case studies, that is, we investigate S3 policies that are implemented already and how those match-up with the local knowledge space indicators outlined in Framework 2 highlighted above. The regional examples are the Italian regions of Umbria, Valle d’Aosta and Piemonte. For demonstrative reasons, three regions of the same country were chosen to show that even within the same national context, which might indicate shared national economic social and institutional structures, one can observe a variety of diverging S3 policy approaches. Furthermore, those specific examples replicate the three general patterns that were identified across all the regions included in the analysis.^5^

Specifically, we attempt to highlight how regions address their current S3 policies in the context of the framework presented in Figure 5 by plotting all technology sectors onto the two-dimensional plane, while subsequently also highlighting those technological knowledge domains that are currently directly addressed by a region’s current S3 policies. The three examples serve as a comparison of three different approaches to S3 policies, which essentially sets the geographical aspect, the degree of regional innovation development, and the institutional setting in context with the framework developed here. In each of the subsequent figures solid triangles indicate where the regional S3 policy is directed towards local knowledge domains in each of the three examples.

**Figure 6.**
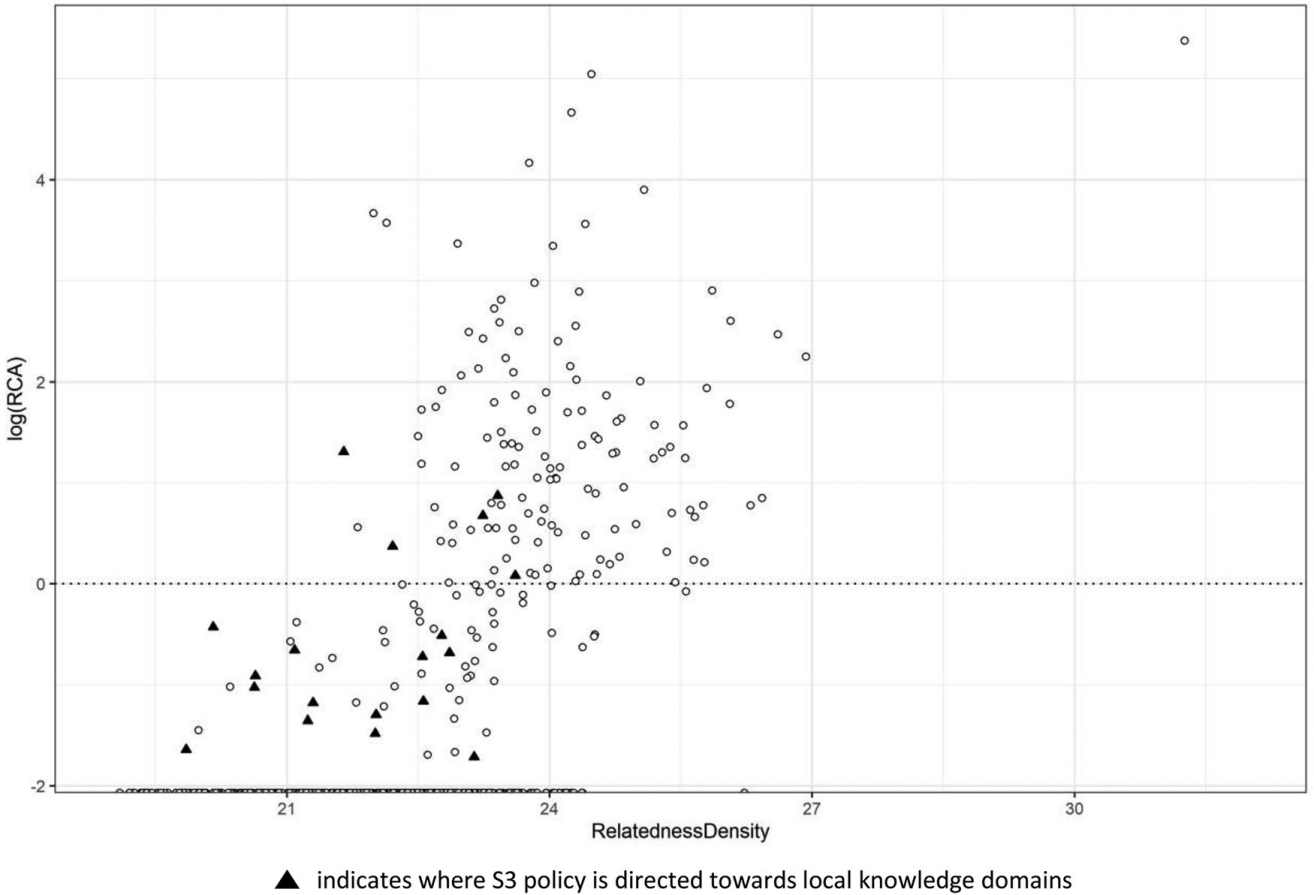
Framework 2: Revealed comparative advantage (RCA) and relatedness density of Umbria.

The first case (Figure 6) is Umbria, a medium-sized Italian region, with several industrial districts and with a fertile ground for the development of business and new economic sectors. Figure 6 shows that the region chose to direct its S3 policy action only towards a limited number of sectors, that is, knowledge domains highlighted by a solid triangle, but with a higher prevalence of policies implemented towards potential domains rather than to those already specialised, that is, knowledge domains that are below the RCA > 1 threshold line. This approach might represent a virtuous example of a medium-sized European region, which decided to exploit the synergies between potential and specialised domains, paving the way for opening up new paths for development, which in turn might benefit its diversification process and subsequent economic growth. Umbria’s approach is implemented in a context where the industrial setting has always been present and active and therefore, the institutional and organisational strengths acquired incentivise to target emerging sectors, which through the well-established network within the districts might easily get specialised in the future, and thus even get more embedded in the local knowledge space. It is believed that these factors might play a crucial role for the innovative process, providing capabilities and fertile ground for entrepreneurs to develop new growth paths and exploit existing strengths. Here, the S3 policy design that is implanted should, by definition, result in the growth of ‘potential’ sectors via the development and the promotion of knowledge-intensive start-ups and research spin-off initiatives, ultimately leading to a more diversified network of sectoral specialisation.

Similar patterns have been identified also in Abruzzo and Sardegna.^6^ Indeed, this strategy might be optimal for similar intermediate regions, which already have some specialised sectors at the foundation of their economic structure but also a strong organisational structure as well as previous experiences of industrial cooperation, all of which might be essential in order to ensure support for sectors that have the potential to reach a comparative advantage in the future. Essentially, following this strategy approach should enable those regions to invest in a diversified portfolio of sectors, subsequently triggering their economic growth through network dynamics and interactions within local actors. Subsequently, such processes, which are aligned with the foundational ideas of the EDP, will generate opportunities to collaborate with actors pursuing similar specialisations in other regional settings, something that further enables regional branching opportunities (Kogler et al., 2023b).

**Figure 7.**
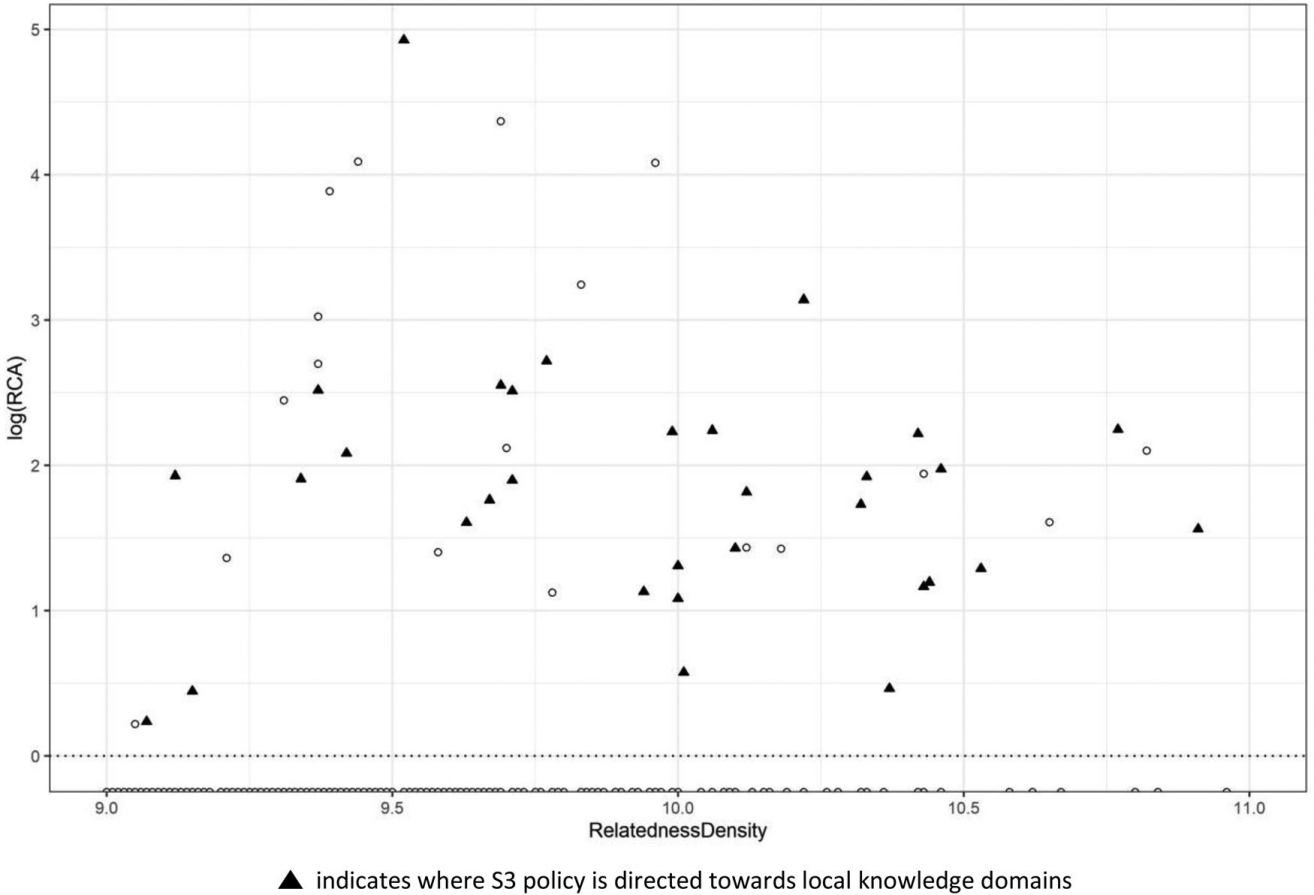
Framework 2: Revealed comparative advantage (RCA) and relatedness density of Valle d’Aosta.

The next example (Figure 7) depicts the local knowledge space of Valle d’Aosta, the smallest of Italian regions. It is exemplary for rural and peripheral regions, also due to its mountainous setting, that feature a small economic and industrial structure based on fewer sectors but also a higher degree of functional specialisation. It is evident from the sectors that are covered by the current regional S3 policy action (domains highlighted with solid triangles in Figure 7) that Valle d’Aosta adopted. It is a policy strategy that almost exclusively focuses on already specialised knowledge domains. For such a small region, the strategy to reinforce existing pattern of specialisation might be optimal since it may not be feasible to find resources to invest in potential or new sectors due to the small size of the regional economy and perhaps associated limited innovation opportunities and capabilities. In this scenario, building on the already embedded knowledge domains and then to reinforce them might be the best and least risky approach. In this specific case, S3 implementation has focused on information and communication technologies (ICTs) as well as the further development of technologies for manufacturing, mountain tourism and solutions for more sustainable productions and the building of smart mountain cities and communities. We found similar patterns of S3 policy actions across other small Italian economies, for example, Molise and Basilicata.

Indeed, for small and less-developed regions, informal coordination mechanisms are often crucial for the regional innovation process, but it might also be challenging to work in collaboration with stakeholders and vested interest players, which may have different objectives. Therefore, it is usually the preferred strategy to reinforce and build up the existing knowledge domains, as investing in the potential ones might only disperse scarce resources towards sectors with weak industrial, organisational and institutional structures. However, this kind of approach might also represent a missed opportunity for small and less-developed regions, perhaps even a risk considering that the reinforcement of existing structures might also be at the root of potential regional lock-in resulting in overall decline. Considering that, investments towards the development of an institutional framework able to improve regional capabilities and to enhance innovative entrepreneurship might be crucial in order to widen the regional diversification strategy and trigger further innovation for sustained growth and prosperity.

**Figure 8.**
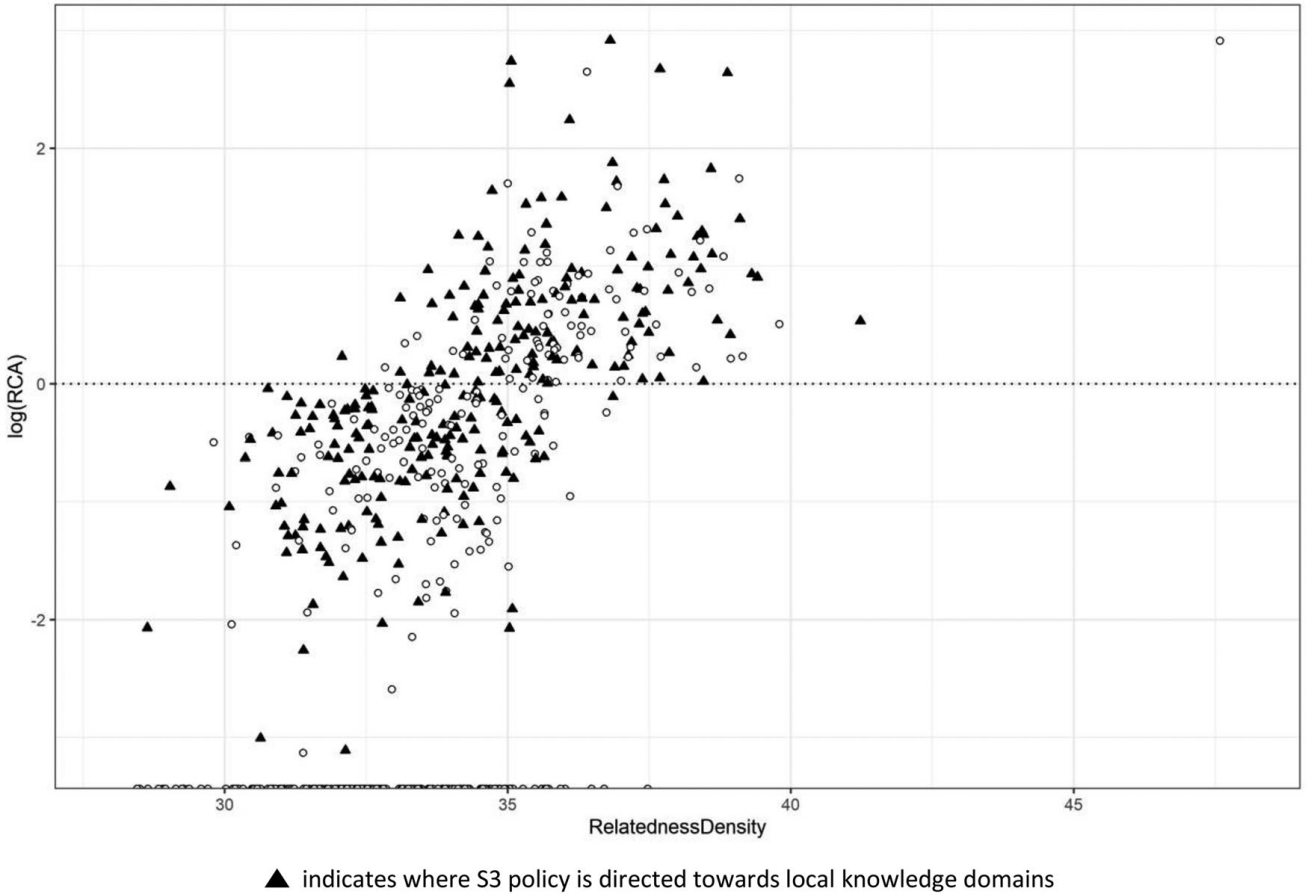
Framework 2: Revealed comparative advantage (RCA) and relatedness density of Piemonte.

In our final example, Figure 8 indicates the local knowledge space of Piemonte. This is one of the most developed of Italian regions, with many diverse industrial sectors comprised of a variety of small, medium and large economic entities representing different levels of specialisation, a truly industrial hub. It is clear that the S3 policy action in this region is directed at many, both already specialised and also potential, technological knowledge domains. Furthermore, it is also evident that there is a much greater presence of technology classes with respect to our previous two examples of Umbria and Valle d’Aosta. The well-defined and mature industrial structure enables this region to adopt a mixed strategy with the objective to support those domains that hold the potential for future diversification and specialisation, while also directing resources towards the strengthening of those sectors that are already well established and embedded in the local knowledge space. A detailed evaluation reveals that the implemented S3 policy focuses on such sectors as biochemical and automotive industry, but also on knowledge relevant for innovation in the textile, fashion and food industry, or technologies for health and well-being, aerospace and mechatronics.

The example of Piemonte and its implemented S3 policy is quite representative for well-developed regional economies that rely on advanced capabilities in the designing of innovation strategies and policies, where both the industrial and the institutional structures are well-equipped to set priority areas, combining strategies for both specialised and non-specialised sectors. Indeed, similar patterns, although sometimes to a varying degree and focus, are also observed in comparable Italian as well as other European regions, including Emilia-Romagna, Toscana, Liguria and Lombardia. This particular approach to S3 policy design is ideal to boost the overall and widespread development and growth of the regional economy by simultaneously upgrading existing and foundational industrial sectors while also investing in the emerging sectors to ensure a further diversification of the regional economy in the future. However, such a strategy also goes well beyond the foundational principles of S3 and thus somewhat represents a more all-inclusive regional innovation policy approach.

In summary, the results and insights derived from the three examples presented here show that regional-specific S3 are frequently guided by very different objectives and approaches when observed across the heterogenous collection of regional economies, and that they might heavily depend on the present regional framework, as well as on place-based economic, institutional, and geographical contexts. Pending on a region’s capabilities and available resources, the EDP might take different routes. Umbria, a medium-sized region, addresses its objectives by focusing on potential knowledge domains and relying on a well-connected industrial setting. Here, the EDP focuses on the development of emerging sectors, taking advantage of the potential synergies between emerging and specialised economic activities. Valle d’Aosta, a small and less developed region, on the other hand reinforces pre-existing patterns of specialisations. This region concentrates its resources concerning S3 policy implementation on already specialised knowledge and innovation activities. Given resource constraints, limited system capacities and a lack of opportunity in terms of potential domains that are available, this might be the most sensible approach here; however, failure to invest in the EDP, and here especially in the institutional and entrepreneurial aspects of the innovation process, might impede the development of new capabilities and eventually result in lock-in that will be difficult to escape in the future. In contrast, as in the case with Piemonte that represents a much larger and more advanced economy, following a mixed approach in terms of policy actions that focus on new sectors that hold a high potential of diversification as well as on upgrading already specialised sectors offers of course ample opportunities. Here, the EDP can rely on a strong foundation of institutional entrepreneurship and place-base leadership, and through that it is feasible and within reach to focus on supporting innovation processes amongst already existing patterns of specialisation while also attempting to initiate new development paths. It certainly is not always the case that the much heralded and emphasised vision of economic diversification presents itself as a perfect strategy, especially when regional institutional structures and capabilities are weakly defined and developed. While the evaluation of organisational, institutional and entrepreneurial aspects is of course one very significant factor that needs to be kept in mind when defining a regional innovation strategy, awareness and insights on local capabilities and their relative configuration in the regional knowledge space are certainly of equal importance. The two evaluation and planning frameworks suggested here speak directly to the latter, and thus mainly to only one side of the same coin and even there not without limitations. Nevertheless, it provides an important steppingstone towards applicable and usable tools for the evaluation of regional knowledge spaces, which in turn should provide regions with more detailed insights on their relative strengths in producing and circulating knowledge of economic value and as such much needed intelligence that can feed into the design of effective and successful regional innovation strategies. There are particular policy implications that result from this work, which we will discuss in turn.

## 5. POLICY IMPLICATIONS

The relevant literature available on S3 policy reveals the significant difficulties regions face in the actual implementation of the policy initiative. Frequently, on the onset it is the lack of ability to identify the optimal strategy and to unleash the full potential of their current innovative capabilities to generate future paths of development and growth. However, and equally relevant, it is also the lack of information and indicators to enable entrepreneurs to discover ample opportunities to engage in the discovery process that might lead to new growth paths and branching opportunities that emanate from existing strengths that might provide a springboard to transition into new spheres of the local knowledge space.

The first policy implication of this work is therefore to provide regions with the urgently required tools they need to properly follow the prescribed EDP, which will enable them to choose the innovation path that matches their local knowledge stock, and even more importantly the specific configuration thereof. The framework we provide here would give regions the means to overcome the ‘one size fits all’ and broadness of the S3 concept and enable them to access their technological knowledge production system, which combined with their institutional and economic context can actively help them pursue the EDP via the identification of those areas that should be prioritised among their already available knowledge pool. It is therefore key for regions to directly invest in the development of skills that are required to properly use this tool, which in turn should empower them to manage S3 policy implementation more efficiently.

The second policy implication emerging from this work is that each region follows its own EDP. The optimal innovation strategy across regions might differ considerably pending on the strengths and weaknesses in the regional knowledge space and its ability to create opportunities for new innovative paths. Therefore, diversification in new or emerging sectors might not always be the best strategy for each region. It may be preferable, in particular for smaller and lagging regions that are lacking sufficient synergies for new activities, to pursue a strategy that aims to strengthen existing and specialised sectors instead of directing resources towards the discovery of new activities. However, these choices may change over time and therefore having an accessible tool that provides applicable and ready-to-use information as part of the strategy, which also considers emerging innovative development paths along the way, is essential. It would support a long-term planning process whereby a region could decide to invest into an emerging sector, one that might currently have little prospect giving the lack of synergies that can be leveraged in the local knowledge space, to create opportunities through transition and/or modernisation mechanisms that in turn initiates a repositioning of their local knowledge space, where leveraging of capabilities, in line with the suggested framework, actually becomes feasible. To reiterate, both the industrial composition and a structure for creating new opportunities for entrepreneurship are crucial for the innovation process and can lead to different, diverging, regional paths (Grillitsch & Asheim, 2018), well beyond simple evolutionary considerations (Kogler et al., 2023a). As Barzotto et al. (2020) point out, regions need to be able to design their S3 policy in line with their strengths and unique potential, giving also struggling regions the opportunity to find their innovation road-map; Policies should provide necessary resources for all regions to enhance their own capabilities and innovation path.

The third policy implication of this research supports the need to create new innovation opportunities via novel combinations of already present knowledge and expertise (Grillitsch & Sotarauta, 2020; Schumpeter, 1911). Sharing knowledge across different sectors can gather useful insights from past experiences and can help readdress innovation paths in novel ways. In this sense, also public–private partnerships and extra-regional relationships should be enhanced, as they can support the innovation process through the development of capabilities and the identification of new resources for investments (Kogler et al., 2023b). Bringing together different stakeholders, such as local actors and firms, can help identify the technological gaps, allow for the development of new ideas, and identify new potential ways for innovation, all through the cross-fertilisation of ideas and leveraging synergies among partners. The combination of regional and international networks through collaborations can enhance the interactive learning process and the innovation process (Tödtling & Grillitsch, 2015). Indeed, extra-regional sources and collaborations can give regions access to complementary knowledge that can compensate the existing knowledge pool and enable the diversification process (Kogler et al., 2023b). The access to these extra-regional sources might depend on several factors related to the regional innovation systems and to the firms’ knowledge (Tödtling & Grillitsch, 2015). However, although most of the studies found that it should be easier for those regions that are already embedded in global innovation networks, also peripheral regions tend to pursue external collaborations, to compensate for the lack of opportunities to access local knowledge spillovers (Grillitsch & Nilsson, 2015). The opportunity to access extra-local knowledge in order to initiate branching processes that might be limited due to the lack of local synergies in the knowledge space should be considered in the S3 policy design of lagging or small regional economies, in particular as it will make advanced knowledge more accessible due to network effects rather having to carry the burden of immense, and most likely unfeasible, direct investments necessary for paving a way towards unrelated diversification (Barca et al., 2012; Kogler et al., 2023b; Trippl et al., 2009). Further, Abbasiharofteh et al. (2023) highlight that it is especially the links between specialised communities across regional settings that generate novel innovative solutions that deviate from mainstream knowledge recombination trajectories, and as such, identifying and connecting with optimal partners is yet another challenge as well as opportunity to further enhance regional entrepreneurial discovery processes. For a practical application on how to identify optimal regional partners for collaborations that offer ample synergies and learning prospects, see the recent study by Calignano et al. (2024) on EU Framework Programme collaborations in the field of nanotechnology. It is at this intersection where the complementarity of perhaps already present S3 policy design elements and our current suggested framework and tool is most pronounced, and where regions can build and leverage a promising approach in their quest to enhance future generations of regional S3 policy designs and actions, that is, the toolset provides foremost an opportunity to evaluate regional opportunities in the knowledge space, while at the same time also enables regional actors to identify optimal non-regional collaborators and/or competitors.

## 6. CONCLUSIONS

Despite the challenges and potential shortfalls of the concept (Hassink & Gong, 2019; Marques & Morgan, 2018), S3 at their core offer essential valuable ideas and avenues for regions to enhance their regional innovative capacities and economic growth prospects via diversification and investment in promising new knowledge domains and associated innovation. However, regions do face considerable difficulties in implementing successful strategies, frequently due to the lack of skills and capabilities, because the institutional quality might not be adequately equipped to convert a vision into reality, or, and perhaps more pervasive, because of the absence of applicable and easy-to-use methods and tools that would support regions in the decision-making process on where to situate priorities in order to target the most central and potential capabilities in their regional innovation system. The analytical approach proposed here, along with the findings provided, offer ample insights into how present regional S3 policies can be evaluated according to their fit in terms of present knowledge production capabilities, but equally important, also points towards an approach for more direct and efficient policy actions in future S3 endeavours.

The first framework provides an overview of how to assess the position of regions that have implemented an S3 in terms of their priorities towards knowledge domains that have either a high connectivity or brokerage power within their respective knowledge space. Findings indicate a sort of ‘all or nothing’ approach across the regional S3 policies that are currently implemented, as they either target domains that rank on average high or low on both indicators with fewer examples where we observe a mixed strategy. While this study only provides an aggregate overview, the framework provided enables each region to assess its detailed knowledge space, and thus to evaluate the connectivity and brokering power of each and every node in the network, which in turn facilities the design of its S3 policy accordingly.

Employing the second framework, we showed a variety of S3 designs that are implemented across regions. The spectrum ranges from policy designs that largely target already regional specialisations, and thus show little to no ambitions to develop new capabilities outside of present strengths, to those where the focus is directed at somewhat weakly embedded knowledge domains that might have no chance to become specialised capabilities in the local knowledge space in the short term. In a third scenario we highlighted an example where the implemented S3 pursue a mixed approach, that is, based upon knowledge that has the potential for future diversification, but also that is already embedded in the local knowledge space. Observing those patterns indicates that there is not a single S3 for each regions, but rather a diverse variety of approaches that are currently utilised. Further, it heavily depends on what kind of S3 policy design is the most suitable, taking into account that the objective is to leverage opportunities in a current knowledge space, while balancing potential significant limitations and resource constraints. Accordingly, this piece of research, including the frameworks and tools, provides valuable insights well beyond local technological capabilities as indicated by technical knowledge domains via patent documents.

The literature that investigates the coherence between S3 implementation and the regional knowledge and technology space (e.g., Di Cataldo et al., 2022; Gianelle et al., 2020; Marrocu et al., 2023) points toward the importance of the institutional quality as an enabler of S3 implementation, and also shows how alternative indicators, for example, employment data, can be used to construct regional spaces of economic activity. Our contribution to this strand of literature is to provide an accessible tool that takes a more focused approach on knowledge production capabilities of a specific region, and that enables a case by case assessment of how S3 policies across Europe have been implemented. Our aim is to overcome the general insights of empirical findings that are presented in the relevant literature, and to recommend an avenue that can help to evaluate the different strategies that already exist, while simultaneously encouraging a more in depth discussion on the proper development of regional innovation policies based on a better understanding of present regional existing strengths, as well as their future potential in order to initiate or reinforce regional innovative processes. Essentially, the tool that is offered here provides the opportunity for policymakers to encompass different ways to measure the fitness and adequacy of their particular regional S3 strategy in the present and the future. There is one limitation of our tool, it does not provide a perspective of the trinity of agency in the region. The properties of regional-specific institutional settings and the innovative entrepreneurship as well as place-based leadership potential needs to be assessed on a case by case basis and in a systematic way, something that is beyond the capability of the toolset that is provided here.

In terms of future research directions and potential avenues to expand the proposed framework and associated toolset, there are plenty of opportunities. The implementation of the concept of ‘entry-potential’ (Kogler et al., 2022) would be one logical extension that would also consider the life cycle perspective of particular knowledge domains. Essentially, two domains that have similarly high values in terms of their knowledge space centrality measures, but with an RCA < 1, and therefore would be identified as potential candidates for S3 policy intervention, might exert a very different degree of influence on the future configuration of the regional knowledge space. This is something that needs to be considered in the decision-making process when it comes to prioritising sectors for future S3 initiatives. In terms of expanding the online tool^7^ it would be most effective to offer additional information on the individual node properties. For example, the number of entities and inventors that were directly involved in the production of specific knowledge domains could prove an essential piece of information when making investment decisions. These insights would be especially important for smaller regional economies where frequently there are very few entities and individuals contributing to single domains or even the entire knowledge space. On the other hand, this would also provide the opportunity to directly engage with knowledge producing actors more directly, which in turn would enable a more engaged entrepreneurial discovery process.

Notwithstanding the potential expansion noted above, the approach outlined in this research will certainly have a positive effect on the EDP that is at the core of S3 policymaking, and it is anticipated that it will assist regional decision makers when choosing the most relevant knowledge domains to prioritise for their unique S3. Providing a comprehensive perspective on the regional knowledge space, this tool will also raise more awareness of the different opportunities regions have for readdressing their present development paths, or acquiring new knowledge by engaging in external collaborations, which in turn will help them to in their quest to achieve advanced technological innovation outcomes that will also enhance their regional economic growth prospects.

## Supplementary Material

Supplemental Material

## Data Availability

The data that support the findings of this study are available from the European Patent Office (EPO) via its PATSTAT database (https://www.epo.org/en/searching-for-patents/business/patstat); and from the Smart Specialisation Platform of the European Commission (https://s3platform.jrc.ec.europa.eu/home).

## NOTES

1. The rKnowledge Dashboard offers an opportunity to investigate the unique regional Knowledge Space and associated measures of specialisation, connectedness and centralities for 685 European and 541 North American metro and non-metro economies. For the rKnowledge Dashboard, see https://www.rknowledge.org/.

2. See https://awekim.shinyapps.io/SS_shiny_R/.

3. For further details, see https://s3platform.jrc.ec.europa.eu/home. Sörvik and Kleibrink (2015) provide further insights into the S3 platform.

4. For a more detailed discussion of the role of ‘entry-potential’ in the context of a pan-European regional study, see Kogler et al. (2022).

5. For detailed results for all regions according to both frameworks and four indicators, see https://awekim.shinyapps.io/SS_shiny_R/; for insights for European regions that are not included in the present analysis, see https://www.rknowledge.org/.

6. For detailed results that highlight each regional knowledge space and associated measures highlighted in Frameworks #1 and #2 developed here across all European regional economies, see https://awekim.shinyapps.io/SS_shiny_R/.

7. See https://www.rknowledge.org/.
