# Supplementary material for: Smart Specialisation Strategies and regional knowledge spaces: how to bridge vision and reality
Source: Reg Stud. 2024 Jun 6;58(12):2501–17. doi: 10.1080/00343404.2024.2355985 (PMC11614045; doi:10.1080/00343404.2024.2355985)
Supplement: Supplemental Material [file CRES_A_2355985_SM2758.pdf]

Appendix A

Table A1. List of regions included in the overall sample

	NUTS-1	NUTS-2
	BE1, BE2, BE3, DE1, DE2, DE3, DE4, DE5, DE6, DE7, DE8, DE9, DEA, DEB, DEC, DED, DEE, DEF, DEG, NL1, NL2, NL3, NL4, UKD, UKF, UKJ, UKK, UKL, UKM, UKN, UKZ	AT11, AT12, AT13, AT21, AT22, AT31, AT32, AT33, AT34, DK01, DK02, DK03, DK04, DK05, EL11, EL12, EL13, EL14, EL21, EL22, EL23, EL24, EL25, EL30, EL41, EL42, EL43, ES11, ES12, ES13, ES21, ES22, ES23, ES24, ES30, ES41, ES42, ES43, ES51, ES52, ES53, ES61, ES62, ES70, FI19, FI1B, FI1C, FI1D, FR10, FR21, FR22, FR23, FR24, FR25, FR26, FR30, FR41, FR42, FR43, FR51, FR52, FR53, FR61, FR62, FR63, FR71, FR72, FR81, FR82, FR83, FR91, FR92, FR93,FR94, ITC1, ITC2, ITC3, ITC4,ITF1,ITF2,ITF3, ITF4, ITF5, ITF6, ITG1, ITG2, ITH1, ITH2, ITH3, ITH4, ITH5, ITI1, ITI2, ITI3, ITI4, NO01, NO02, NO03, NO04, NO05, NO06, NO07, PL11, PL12, PL21, PL22, PL31, PL32, PL33, PL34, PL41, PL42, PL43, PL51, PL52, PL61, PL62, PL63, RO11, RO12, RO21, RO22, RO31, RO41, RO42, SE11, SE12, SE21, SE22, SE23, SE31, SE32, SE33
Countries	4	11
Regions	31	133
